# IL-17A Mediates Early Post-Transplant Lesions after Heterotopic Trachea Allotransplantation in Mice

**DOI:** 10.1371/journal.pone.0070236

**Published:** 2013-07-30

**Authors:** Philippe H. Lemaître, Benoît Vokaer, Louis-Marie Charbonnier, Yoichiro Iwakura, Marc Estenne, Michel Goldman, Oberdan Leo, Myriam Remmelink, Alain Le Moine

**Affiliations:** 1 Transplantation Medicine, Institute for Medical Immunology, Université Libre de Bruxelles, Gosselies, Belgium; 2 Center of Experimental Medecine and Systems Biology, Institute of Medical Science, University of Tokyo, Tokyo, Japan; 3 Innovative Medicines Initiative, Brussels, Belgium; 4 Erasme Hospital, Université Libre de Bruxelles, Brussels, Belgium; CNRS, France

## Abstract

Primary graft dysfunction (PGD) and bronchiolitis obliterans (BO) are the leading causes of morbidity and mortality after lung transplantation. Reports from clinical and rodent models suggest the implication of IL-17A in either PGD or BO. We took advantage of the heterotopic trachea transplantation model in mice to study the direct role of IL-17A in post-transplant airway lesions. Across full MHC barrier, early lesions were controlled in IL-17A^-/-^ or anti-IL17 treated recipients. In contrast, IL-17A deficiency did not prevent subsequent obliterative airway disease (OAD). Interestingly, this early protection occurred also in syngeneic grafts and was accompanied by a decrease in cellular stress, as attested by lower HSP70 mRNA levels, suggesting the involvement of IL-17A in ischemia-reperfusion injury (IRI). Furthermore, persistence of multipotent CK14^+^ epithelial stem cells underlined allograft protection afforded by IL-17A deficiency or neutralisation. Recipient-derived γδ^+^ and CD4^+^ T cells were the major source of IL-17A. However, lesions still occurred in the absence of each subset, suggesting a high redundancy between the innate and adaptive IL-17A producing cells. Notably, a double depletion significantly diminished lesions. In conclusion, this work implicated IL-17A as mediator of early post-transplant airway lesions and could be considered as a potential therapeutic target in clinical transplantation.

## Introduction

Lung transplantation remains the only therapeutic approach for end-stage lung failure. Although immunosuppressive regimens efficiently control acute rejection [1], two main problems impact recipients’ survival. On the one hand, primary graft dysfunction (PGD) that occurs during the immediate postoperative period is caused by ischemia-reperfusion injury (IRI), and affects up to 25% of the recipients [2]. Clinically equivalent to an acute respiratory distress syndrome (ARDS), PGD is accompanied with an alteration in the PaO_2_/FiO_2_ ratio that may require extracorporeal membrane oxygenation support and remains the major cause of early postoperative morbidity and mortality [2]. Indeed, severe PGD is associated with up to 40% mortality 30-day after transplantation [3]. On the other hand, chronic rejection, namely the bronchiolitis obliterans (OB), is a fibroproliferative disease affecting the small airways. OB leads to an irreversible decline in lung function and accounts for more than 50% of lung allograft failures occurring within 5 years following transplantation [4]. Although no causal relationship linking PGD to OB has been demonstrated, some authors have shown that PGD is associated with a greater risk of OB [5], and histopathologic reports indicate that both inflammation and injury precede an aberrant tissue repair and epithelial regeneration that leads to the fibrous obliteration of the small airways occurring during OB [6].

Production of IL-17A, a pro-inflammatory cytokine involved in autoimmune and infectious diseases, has been observed after heart and kidney transplantation during both IRI [7,8] and graft rejection [9,10]. In lung transplanted patients experiencing PGD or chronic rejection, high amounts of IL-17A were found in the bronchoalveolar lavage (BAL) suggesting its involvement in lung PGD and OB respectively [11,12]. In rodents, studies demonstrated a pathogenic role of IL-17A in an *in situ* lung IRI model [13] and in the generation of OB after lung transplantation across minor MHC mismatch [14]. However, the relative importance of IL-17A mediated tissue damages during the chronological stages of transplant rejection remains poorly understood in case of MHC mismatches. Indeed, cells from both innate (γδ^+^, NK cells, neutrophils) and adaptive immunity (CD4^+^ and CD8^+^ T cells) can produce IL-17A and may therefore generate graft lesions at different time points after transplantation [15].

Herein, we used the heterotopic trachea transplantation (HTT) model described by Hertz et al. [16] in which syngeneic and fully allogeneic tracheas undergo an initial epithelial injury [17]. Subsequently, the syngeneic organs rapidly heal and recover a normal structure by day 28 [17,18]. In contrast, the allogeneic grafts develop an obliterative airway disease (OAD) considered an experimental equivalent of BO. Altogether, this model allowed us to decipher the role of IL-17A in early and advanced post-transplant airway lesions.

## Materials and Methods

### Mice

Wild type C57BL/6 and BALB/C mice were purchased from Harlan, Netherlands. IL-17A^-/-^ C57BL/6 mice were kindly provided by Dr Iwakura (University of Tokyo, Tokyo, Japan). γδTCR^-/-^ C57BL/6 mice were kindly provided by Dr. F. Huaux, Université Catholique de Louvain, Brussels, Belgium. Eight to twelve weeks old animals were used and animals were bred in our specific pathogen-free animal facility. All animals received humane care in compliance with the Principles of Laboratory Animal Care formulated by the National Institute of Health (Guide for the Care and Use of Laboratory Animals, Eighth Edition, National Research Council, 2010) and protocols were approved by the Ethical Committee from the Biopole ULB Charleroi (agreement # LA2500519).

### Heterotopic trachea transplantation

Heterotopic trachea transplantation (HTT) was performed according to an adaptation of the method of Hertz [16]. Briefly, donor mice were euthanized in 100% CO_2_. Donor hearts and lungs were then exposed via a midline incision through the skin and peritoneum extending through the rib cage and sternal notch. Thymus tissue was dissected away. The trachea was separated from the esophagus by blunt dissection, excised from the first tracheal ring to the main bronchi and placed in 0.9% sodium chloride until transplantation. Recipient mice were anaesthetized with a mixture of xylazine (Rompun) 5% and ketamine 10% in phosphate-buffered saline (PBS). After shaving a surface of 0.5cm x 0.5cm over the scapula, a 3mm incision was made through the dermis and a 1.5cm x 1.5cm pouch was created by blunt dissection over the posterior upper back area. One trachea was placed in each pouch. When recipients were co-transplanted, syngeneic and allogeneic tracheas were grafted on opposite flanks. Skin was then closed with 5/0 silk suture. The time between harvesting and transplantation never exceeded 15 minutes. Recipient mice were monitored until full recovery and then every day until sacrifice. At the time of harvesting, recipient mice were sacrificed by cervical dislocation and grafts were removed by blunt dissection.

### Antibody treatments

When specified, recipients received neutralizing or depleting antibody injections. 300µg of the neutralizing anti-IL17A (clone MM17F3) or isotype control (clone CRL16.67, both kindly provided by Dr. C. Uyttenhove, Ludwig Institute for Cancer Research, Brussels, Belgium) was intraperitoneally (IP) injected, starting the day of transplantation and followed by two injections per week after transplantation. 500µg of the depleting anti-CD4 cocktail (clones YTS191 and YTA 3.1.2) or isotype control (clone YCATE) kindly provided by Dr. S. Cobbold, Sir Dunn School of Pathology, Oxford University, UK) was IP injected on days 0, 2 and 4 after trachea transplantation. A single dose of 300 µg of the depleting anti-gammadelta T cells (clone UC7-13D5) or control isotype (clone PARSI 19) was administered IP 7 days before transplantation. Double-depleted animals were treated with both antibodies together. Depletion efficiency was measured by flow cytometry on draining lymph node cells at the time of harvest and reached >95% for both subsets.

### Histopathology

Grafts were harvested at 5, 8 and 28 days after transplantation. Cross-sectional specimens were fixed in 4% formaldehyde, embedded in paraffin, sectioned at 5µm-thickness and stained with haematoxylin-eosin, Masson’s trichrome and PAS. All specimens were examined in blind fashion and scored as previously described [19]. For lumenal occlusion assessment, Masson’s trichrome stained sections were photographed at x40 magnification using a Nikon DXM1200F camera on a Nikon Eclipse 80i microscope. Images were then analyzed with Zeiss AxioVision 4.7 software and surface from the tracheal cartilage and the free lumen were measured. Residual free lumen was calculated as follows: (free lumen/surface at cartilage) x 100. Results are expressed as percentages.

### Immunostaining

Five µm paraffin sections were cut, deparaffinized and rehydrated. Endogenous peroxidase activity was first quenched by H_2_O_2_ peroxidase blocking reagent (DakoCytomation). For CK14^+^ epithelial stem cells staining, sections were then incubated with 1/100 diluted anti-CK14 antibody (clone LL002, Novocastra, UK) for 20 minutes at room temperature. Sections were then washed and incubated with 1/500 diluted goat anti-rabbit antibody (Jackson Immunoresearch, West Grove, PA) for 20 minutes at room temperature (RT). For neutrophil staining, sections were then incubated with 1/50 diluted anti–Ly-6G Ab (BD Pharmingen, San Diego, CA) for 30 min at RT. Sections were then washed and incubated with 1/500 diluted biotinylated goat anti-rabbit Ab (Jackson Immunoresearch) for 30 min at RT. Thereafter, streptavidin-HRP was added and coloration was revealed using diaminobenzidine (DAB) with the substrate chromogen system from Dakocytomation. Two independent operators counted positive cells. For CK14^+^ cells, 3 systematic non-overlapping fields at the ×200 magnification were considered for each tracheal graft. For neutrophils, all Ly-6G^+^ cells on one section were counted.

### RNA extraction and real-time RT-PCR

Total RNA was extracted from trachea transplants using the MagnaPure LC RNA Isolation Kit III for tissue (Roche Diagnostics) according to manufacturers’ instructions. Reverse transcription and real-time PCR were performed using LightCycler-RNA Master Hybridization Probes (one-step procedure) on a Lightcycler 480 apparatus (Roche Diagnostics). Beta-Actin was used as RNA loading control. The primers were custom ordered from Eurogentec as follows: IL-6, forward, 5’-AGGATACCACTCCCAACAGACC-3’, reverse, 5’-AAGTCCATCATCGTTGTTCATACA-3’ and probe, 5’-FAM-CAGAATTGCCATTGCACAACTCTTTTCTCA-TAMRA-3’; IFN-γ: forward 5’-GGATGCATTCATGAGTATTGC-3’, reverse 5’-GCTTCCTGAGGCTGGATTC-3’ and probe 5’-FAM-TTTGAGGTCAACAACCCACAGGTCCA-TAMRA-3’; IL-17A: forward 5’-GCTCCAGAAGGCCCTCAG-3’, reverse 5’-CTTTCCCTCCGCATTGACA-3’ and probe 5’-FAM‑ACCTCAACCGTTCCACGTCACCCTG‑TAMRA‑3’; IL‑1β: forward 5’-CAACCAACAAGTGATATTCTCCATG-3’, reverse 5’-GATCCACACTCTCCAGCTGCA-3’ and probe 5’-FAM‑CTGTGTAATGAAAGACGGCACACCCACC‑TAMRA‑3’; TNF-α: forward 5’-TCTTCTCGAACCCCGAGT-3’, reverse 5’-CCTCTGATGGCACCACCAG-3’ and probe 5’-FAM‑TAGCCCATGTTGTAGCAAACCCTCAAGCT‑TAMRA‑3’. HSP70 primers were purchased as ready-made mix from Applied Biosystems (cat# Mm03038454_S1). Graft mRNA levels are expressed as 2^-ΔΔCT^ in which CT represents “cycle of threshold”, ΔΔCT= ΔCT_native trachea_-ΔCT_control or anti-IL17 treated trachea_ and ΔCT= CT_gene of interest_ - CT_β-actin_.

### Flow Cytometry and graft-infiltrating cell isolation

To isolate graft-infiltrating lymphocytes (GILs), harvested tracheas were minced and incubated at 37°C for 100 minutes with type I collagenase at 2 mg/mL (Sigma) in a phosphate buffered solution. Intracytoplasmic staining was then performed after cell incubation with 50 ng/mL PMA and 500 ng/mL ionomycin for 4 h with brefeldin A (10 µg/mL) in the last 2 hours; then the cells were incubated for 10 minutes with Fc block, stained for surface markers for 20 minutes, washed with Phosphate Buffer Saline/Bovine Serum Albumin 0.1%/NaN _3_0.01%, fixed with CytoFix/CytoPerm (BD Biosciences), permeabilized with Perm/Wash buffer (BD Biosciences) and labelled with anti-IL17A antibody. Cytometry analysis was performed on a CyAn-LX cytometer using Summit 4.1 software (DakoCytomation).

Pacific-Blue (PB) conjugated anti-mouse CD3ε (clone 500A2), Phycoerythrin (PE) conjugated anti-mouse CD4 (clone RM4-5), anti-mouse γδ-TCR (clone GL3) and anti-mouse H-2K^b^ (clone AF6-88.5), Fluorescein Isothiocyanate (FITC) conjugated anti-mouse CD4 (clone RM4-5), Peridinin-chlorophyll-protein complex (PerCP) conjugated anti-mouse CD3ε (clone 145-2C11) and anti-mouse NK1.1 (clone PK136), AlexaFluor647 anti-mouse CCR6 (clone 140706) and anti-mouse CD16/CD32 (Fc block, clone 2.4G2) monoclonal antibodies and isotype controls were purchased from BD Pharmingen. APC conjugated anti-mouse IL-17A (clone eBio17B7) was purchased from eBiosciences.

### Statistical analyses

Statistical analyses of differences between groups were performed using the two-tailed Mann-Whitney nonparametric test. p<0.05 is considered statistically significant.

## Results

### IL-17A deficiency does not prevent OAD

**Figure 1:**
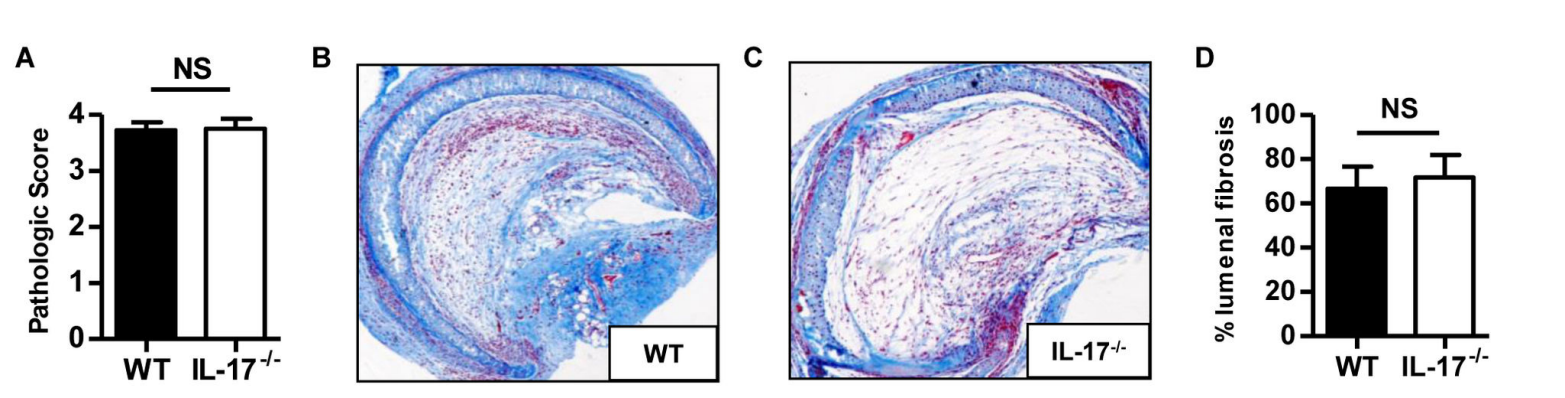
IL-17A deficiency does not prevent obliterative airway disease. Fully allogeneic BALB/C tracheas were heterotopically grafted into WT or IL-17A^-/-^ C57BL/6 recipients and harvested after 28 days. **A**, pathologic scores. **B** & **C**, histologic analysis of allografts harvested from one representative WT and IL-17A^-/-^ recipient, respectively. Masson’s trichrome staining of a whole section (magnification x40). **D**, percentages of lumenal fibrosis. The bars represent the mean ± SEM of 12-15 organs in each group. Data are pooled from three independent experiments.

Heterotopic trachea transplantation (HTT) was performed as described by Hertz et al. [16]. We first addressed the effect of IL-17A on the development of post-transplant obliterative airways disease (OAD). Therefore, fully allogeneic BALB/C tracheas were grafted into C57BL/6 WT or IL-17A^-/-^ recipients. Animals were sacrificed at day 28 post-transplantation. Pathologic score was based on four easily identifiable processes: leukocyte infiltration, loss of the pseudostratified airway epithelial architecture, subepithelial fibrosis, and luminal obliteration due to granulation tissue formation and/or fibrosis, as described in materials and methods. We found that IL-17A deficiency had no final impact on obliterative lesions as grafts harvested from IL-17A^-/-^ recipients exhibited the same pathologic score as observed in grafts from WT recipient mice (Figure 1A). Indeed, the respiratory epithelium vanished in both groups and leukocyte infiltration was comparable. Dense collagen deposits enlarged the lamina propria, thickened the basal membrane and obstructed the lumen (Figure 1B & D). To confirm these results, we also compared lumenal obstruction (as described in materials and methods). We found no differences between WT and IL-17A^-/-^ recipients, which displayed 66 ± 10% vs 71 ± 10% obstruction, respectively (Figure 1C). A network of neovessels underlined these fibro-obliterative processes. IL-17A deficiency alone is thus ineffective in preventing late rejection of fully allogeneic trachea transplants.

### IL-17A deficiency prevents early allograft lesions

**Figure 2:**
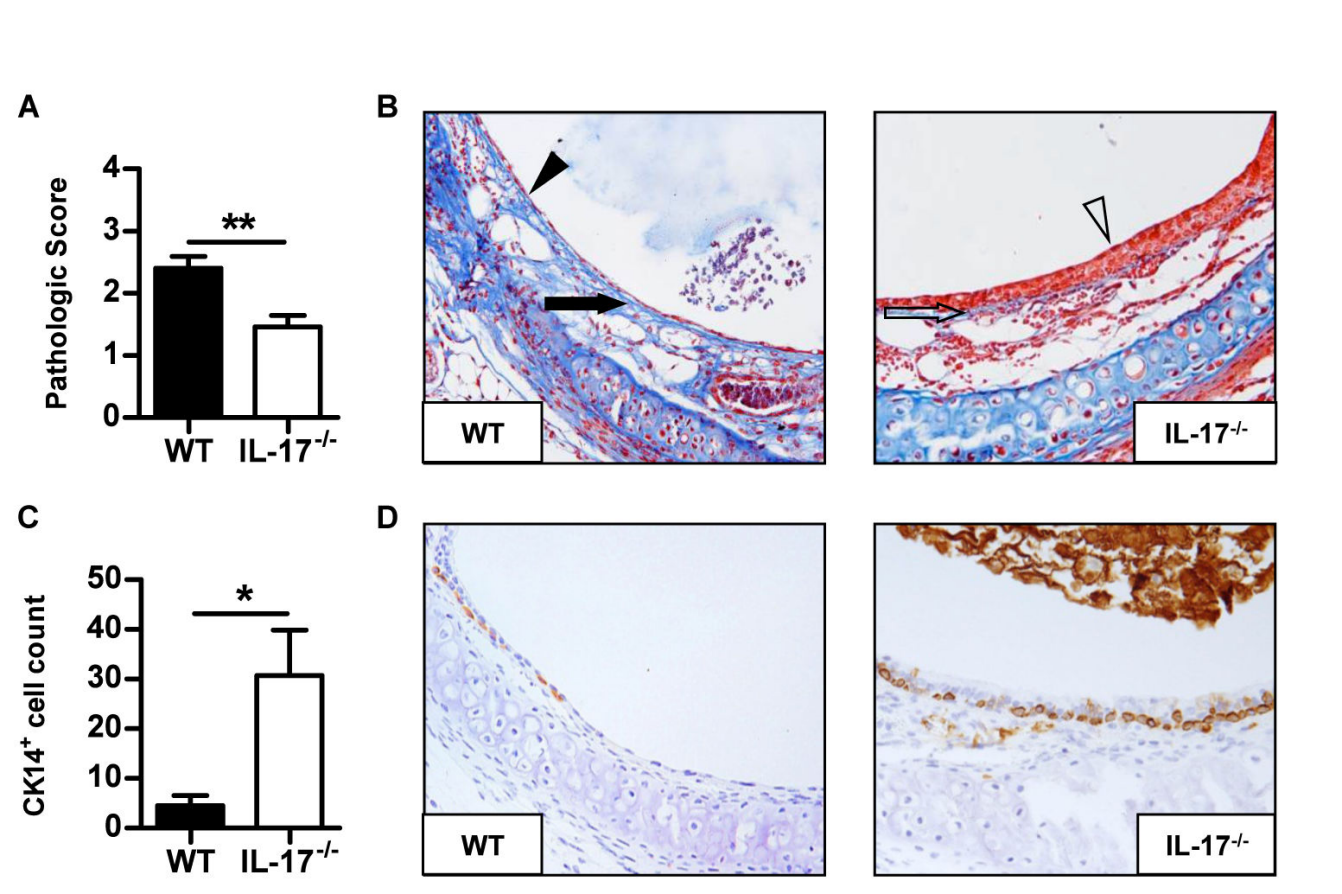
IL-17A deficiency prevents early allograft lesions. Fully allogeneic BALB/C tracheas were heterotopically grafted into WT or IL-17A^-/-^C57BL/6 recipients and harvested after 8 days. **A**, pathologic scores. **B**, histologic analysis of allografts harvested from one WT and one IL-17A^-/-^ recipient, respectively. Masson’s trichrome staining of a whole section (magnification x200). The black arrow shows the basal membrane thickening and the black arrowhead identifies epithelial flattening. The empty arrow shows the normal basal membrane and the empty arrowhead identifies the pseudostratified respiratory epithelium. **C**, CK14^+^ basal cell count after specific immunostaining. **D**, a representative picture of a section stained for CK14 in organs harvested from one WT and one IL-17A^-/-^ recipient, respectively. The bars represent the mean ± SEM of 15 organs in each group. Data are pooled from three independent experiments. *, p < 0,05 and **, p < 0,005.

Because IL-17A can be involved in early inflammatory mechanisms, we next assessed the impact of IL-17A deficiency in early post-transplant lesions. To this aim, BALB/C tracheas were transplanted into WT or IL-17A^-/-^ C57BL/6 recipients for 8 days. As shown in Figure 2A, grafts harvested from IL-17A^-/-^ recipients showed significantly fewer lesions compared to grafts from WT recipients. Indeed, protected organs retained a pseudostratified epithelial architecture and showed little collagen deposits, in contrast to grafts from WT recipients which developed epithelial flattening, basal membrane thickening and subepithelial fibrosis (Figure 2 B). Grafts from IL-17A^-/-^ recipients also preserved a substantial number of CK14^+^ basal epithelial stem cells, which are capable of renewing the whole epithelium after injury [20] (Figure 2 C and D). In contrast, these cells nearly completely vanished in grafts from WT recipients. These experiments demonstrate that IL-17A is implicated in early post-transplant airway lesions.

### IL-17A neutralisation prevents early post-transplant lesions independently of allorecognition

**Figure 3:**
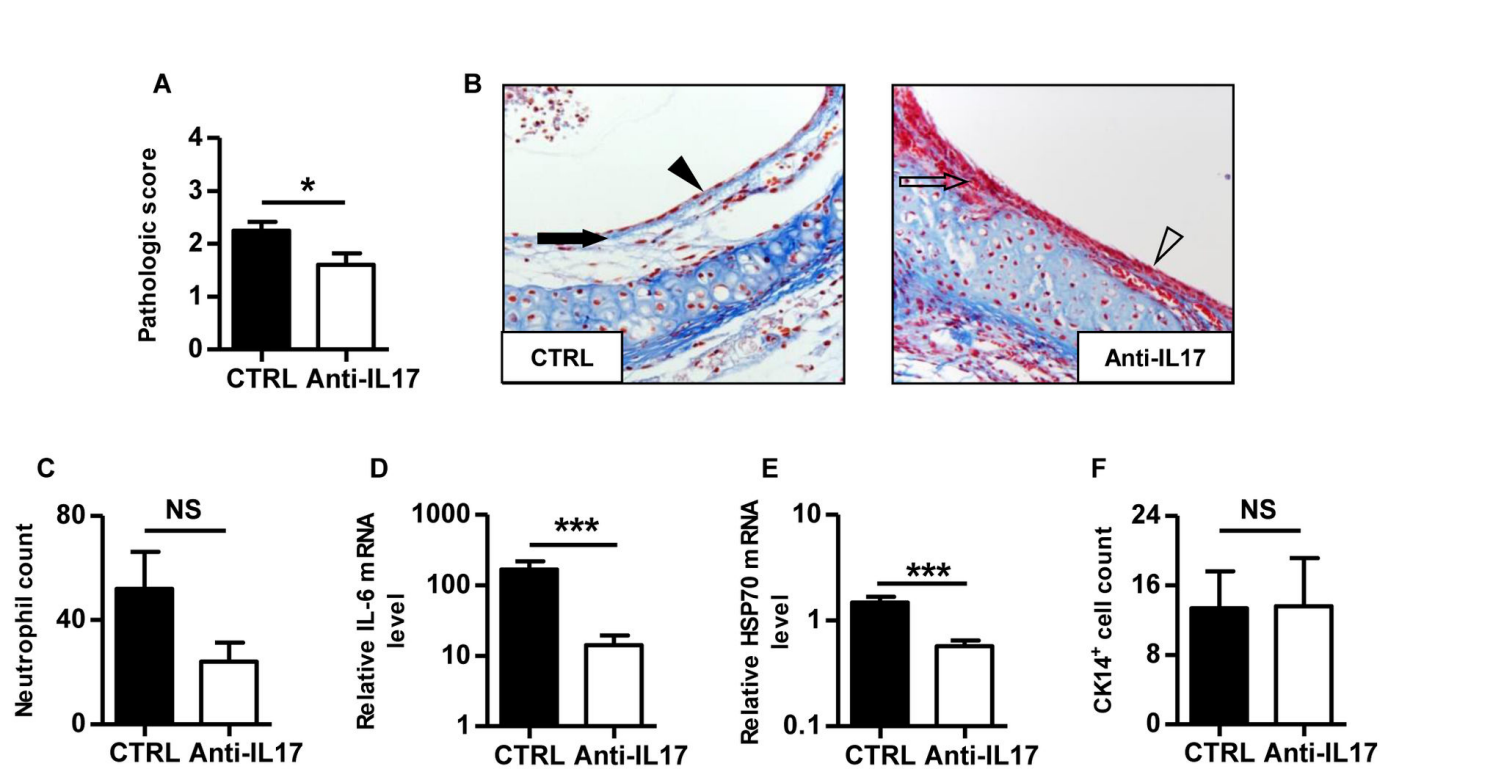
IL-17A blockade prevents early post-transplant airway lesions in syngeneic grafts. Syngeneic BALB/C tracheas were heterotopically grafted into control or anti-IL17 treated BALB/C recipients and harvested after 5 days. **A**, pathologic scores. **B**, histologic analysis of allografts harvested from one control and one anti-IL17 treated recipient, respectively. Masson’s trichrome staining of a whole section (magnification x200). The black arrow shows the basal membrane thickening and the black arrowhead identifies epithelial flattening. The empty arrow shows the normal basal membrane and the empty arrowhead identifies the pseudostratified respiratory epithelium. **C**, absolute neutrophil count after specific Ly-6G immunostaining. **D**, IL-6 mRNA. **E**, HSP 70 mRNA. **F**, CK14^+^ basal cell count after specific immunostaining. The bars represent the mean ± SEM of 10 organs in each group. Data are pooled from two independent experiments. ***, p < 0,001.

**Figure 4:**
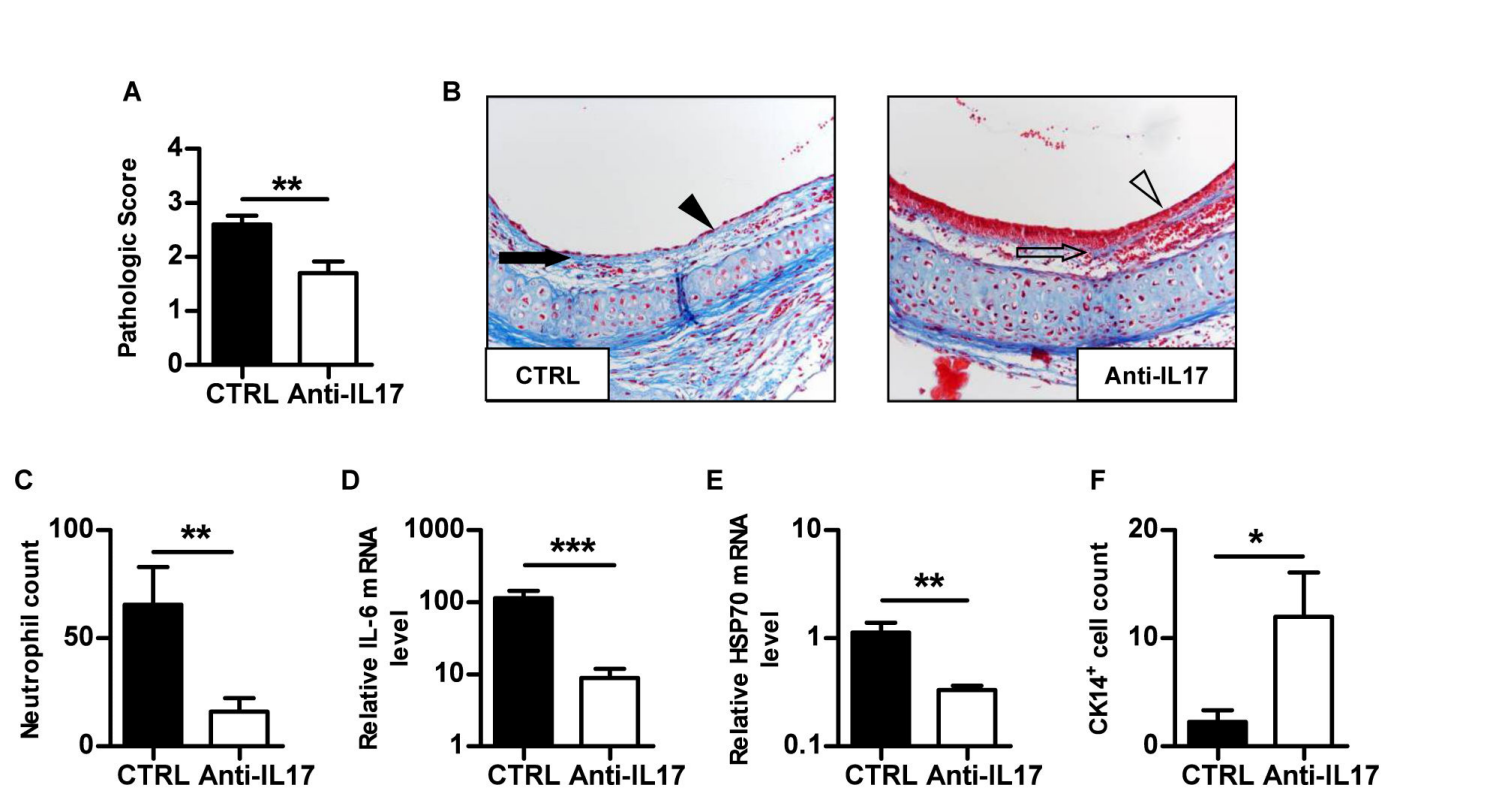
IL-17A blockade prevents early post-transplant airway lesions in fully allogeneic grafts. Fully allogeneic C57BL/6 tracheas were heterotopically grafted into control or anti-IL17 treated BALB/C recipients and harvested after 5 days. **A**, pathologic score. **B**, histologic analysis of allografts harvested from control and anti-IL17 treated recipients, respectively. Masson’s trichrome staining of a whole section (magnification x200). The black arrow shows the basal membrane thickening and the black arrowhead identifies epithelial flattening. The empty arrow shows the normal basal membrane and the empty arrowhead identifies the pseudostratified respiratory epithelium. **C**, absolute neutrophil count after specific Ly-6G immunostaining. **D**, IL-6 mRNA. **E**, HSP 70 mRNA. **F**, CK14^+^ basal cell count after specific immunostaining. The bars represent the mean ± SEM of 10 organs in each group. Data are pooled from two independent experiments. *, p < 0,05; **, p < 0,005 and ***, p < 0,001.

In order to assess the role of IL-17A in the inflammation resulting from the surgical procedure, we performed experiments in syngeneic condition. To confirm our previous results and to rule out a possible strain effect, allogeneic tracheas were grafted on the same recipient background. Anti-IL17 or control antibody-treated BALB/C mice were thus transplanted with a syngeneic BALB/C trachea on the left flank and an allogeneic C57BL/6 trachea on the right flank. Because C57BL/6 organs are more sensitive to IRI than BALB/C [21], all grafts were harvested after 5 days. Although data demonstrated increased IL‑17A mRNA in allografts compared with syngeneic transplants (Figure S1A), we found that syngeneic grafts harvested from anti-IL17 treated animals had fewer lesions compared to control antibody treated animals (Figure 3A). Indeed, grafts from treated animals preserved a pseudostratified epithelium and showed little cellular infiltration in the lamina propria whereas control antibody treated grafts experienced epithelial flattening and basal membrane thickening (Figure 3B). IL-17A blockade controlled neutrophil recruitment, as demonstrated after specific anti-Ly6G immunostaining (Figure 3C). The intragraft IL-6 mRNA expression was also lower in the context of IL-17A blockade (Figure 3D). Furthermore, we demonstrated that IL-17A blockade controlled cellular stress, as attested by a reduction of HSP70 mRNA levels in grafts harvested from anti-IL17 treated animals compared to control animals (Figure 3E). Interestingly, we observed similar numbers of CK14^+^ basal cells in both groups (Figure 3F). As shown in Figure 4A, IL-17A blockade also controlled early post-transplant lesions in allogeneic tracheas. Similarly to the syngeneic condition, this treatment prevented the recruitment of neutrophils (Figure 4C) and significantly decreased IL-6 and HSP70 mRNA expression (Figure 4D & E). In contrast to the syngeneic condition, this treatment prevented the destruction of CK14^+^ basal epithelial stem cells. Further cytokine analysis revealed that IL‑17A blockade increased graft IL‑1β and IFN-γ mRNA levels only in the allogeneic condition, whereas TNF-α remained unaffected (Figure S1B, C and D). As IL-17A inhibition prevented lesions in syngeneic as well as allogeneic conditions, these results indicate that IL-17A is a crucial inflammatory mediator in early post-transplant lesions independently of allorecognition.

### Recipient-derived γδ^+^ and CD4^+^ T cells are codominant sources of IL-17A

**Figure 5:**
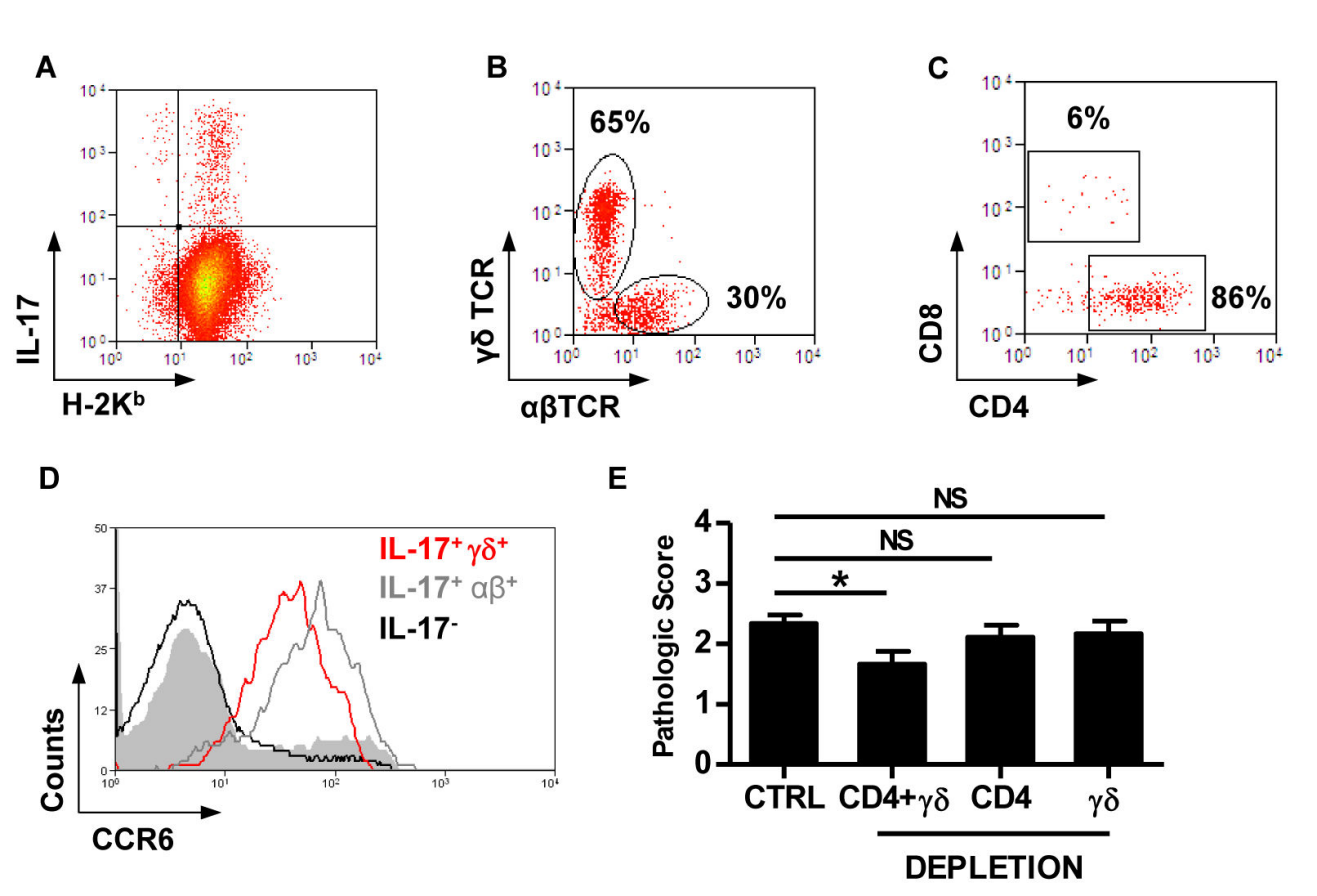
γδ^+^ and CD4^+^ T cells are redundant IL-17A producing T cells recruited into the graft. Flow cytometry and histologic analysis of fully allogeneic BALB/C tracheas grafted into C57BL/6 recipients and harvested after 8 days. **A**, plot representing the intracellular expression of IL-17A in recipient-derived H-2K^b+^ cells. **B**, plot representing the membranous expression of γδ and αβ TCR in IL-17A^+^ H-2K^b+^ GILs. **C**, plot representing the distribution of CD4^+^ and CD8^+^ T cells among αβ^+^ IL-17A^+^ GILs. **D**, plot representing the membranous CCR6 expression in IL-17A^+^ γδ^+^ and IL-17A^+^ γδ^-^ GILs. **E**, pathologic scores of control, combined CD4- and γδ-depleted, CD4-depleted and γδ-depleted recipients, respectively. Each FACS plot is representative of 5 organs and percentages are expressed as the mean of 5 organs. The bars represent the mean ± SEM of n = 6-15 organs in each group.

To characterize the intragraft cellular sources of IL-17A, we transplanted BALB/C tracheas into C57BL/6 recipients for 8 days and analysed the graft-infiltrating cells by flow cytometry. C57BL/6 MHC-I H-2K^b^ specific staining revealed that the majority of the IL-17A-producing cells were recipient-derived (Figure 5A). We observed that γδ^+^ and αβ^+^ T cells represented around 65% and 30% of these cells, respectively (Figure 5B). NK1.1^+^ cells abundantly infiltrated the grafts but did not secrete IL-17A (Figure S2). Further analysis demonstrated that the majority of IL-17A producing αβ^+^ cells were CD4^+^ in contrast to the CD8^+^ counterpart (Figure 5C). Finally, we found that γδ^+^ and αβ^+^ IL-17A^+^ cells had a similar expression of the chemokine receptor CCR6, which is considered specifically expressed on tissue-recruited IL-17A producing cells [15] (Figure 5D).

We next addressed the contribution of each type of IL-17A producing population in our model. B6 recipients of BALB/C tracheas were depleted of CD4^+^ and γδ^+^ T cells either together or separately, as described in materials and methods. As shown in Figure 5E, the lesions were significantly reduced when both subsets were depleted together. However, single depletions of γδ^+^ T cells or CD4^+^ T cells did not prevent lesion occurrence. These results demonstrate that γδ^+^ and CD4^+^ T cells represent the major IL-17A producing cells recruited to the grafts. This also suggests that each IL-17A producing subset can be autonomously pathogenic.

## Discussion

Primary graft dysfunction and bronchiolitis obliterans are the leading causes of morbidity and mortality after lung transplantation. Taking advantage of the HTT model in IL-17A^-/-^ mice, we demonstrated a dominant role of IL-17A in early but not late post-transplant airway lesions. Representing a clinically applicable treatment [22], IL-17A blockade also afforded protection. Interestingly, the protective effect of IL-17A neutralization was observed in both allogeneic and syngeneic conditions, although IL‑17A mRNA levels were much higher in allografts compared with syngeneic transplants. In both cases, the inhibition of neutrophil recruitment to the grafts and the decrease in IL-6 mRNA levels confirmed the impairment of two IL-17A inflammatory downstream mediators implicated in IRI [23,24]. In addition, IL-17A blockade reduced cellular stress as attested by the reduction of HSP70 mRNA levels [25]. In line with this, IL-17A has recently been shown to mediate ischemia-induced cardiomyocyte apoptosis [8]. Our results are in accordance with previous reports demonstrating a role for IL-17A in kidney, liver and lung IRI models in which there is no alloantigen [7,13,26]. In addition, anti-IL17A treatment also reduced the lesions in fully MHC-mismatched allografts. In this setting, IL-17A neutralization allowed the persistence of CK14-expressing airway basal cells witnessing an important feature for epithelial protection. Indeed, these are epithelial stem cells capable of renewing the whole epithelium after injury [20]. Noteworthy, CK14^+^ cells were not affected in the syngeneic condition meaning that epithelial cells are primary allogeneic targets [27]. This difference between the syngeneic and allogeneic conditions strongly suggests there is a synergy of alloantigen-dependent and allo-independent mechanisms of tissue injury.

Innate and adaptive immunity participate in IRI [28]. Cells from both systems can secrete IL-17A [15]. Accordingly, previous reports demonstrated the role of IL-17A producing NKT cells [13] or indirectly implicated Th17 in lung IRI after *in situ* hilar occlusion [29]. In cardiac IRI, γδ^+^ T cells produced IL-17A [8]. In our transplant model, H-2K^b^ expression on intragraft lymphocytes demonstrated that virtually all IL-17A producing cells were recipient-derived graft infiltrating cells. Moreover, we found that γδ^+^ T cells and CD4^+^ T cells, which represent the majority of IL-17A-producing cells, highly expressed CCR6. This chemokine receptor has already been efficiently blocked in a model of rheumatoid arthritis [30] and could therefore represent a potential therapeutic target in IRI. Besides, NK1.1-expressing cells abundantly infiltrated the grafts but did not secrete IL-17A. Although γδ^+^ T cells have been described as stress sensors [31] and played a central role in cerebral IRI [32], tracheas from γδ-depleted or γδ-deficient (data not shown) recipients were not protected against post-transplant IRI. Similarly, depletion of CD4^+^ T cells did not prevent lesions, contrasting with the results from Yang et al. [29]. Concomitant depletion of regulatory T cells may explain this phenomenon [28]. Furthermore, CD4^+^ T cells have been implicated in recovery mechanisms following hind limb IRI [33]. These results implied some redundancy between the innate and adaptive IL-17A producing T cell subsets in mediating early post-transplant lesions, which was confirmed by the beneficial impact of a combined depletion of both subsets.

Interestingly, IL-17A blockade significantly increased IL‑1β and IFN-γ mRNA production only in allografts and not in syngeneic condition. Nevertheless, the role of these cytokines in our early model remains undetermined. Besides, in a model of late fibro-obliterative airway lesions, we recently showed that IFN-γ deficiency enhanced graft damages, supporting an unexpected regulatory role of the cytokine [18]. Further investigations are required to address the complex regulation of these cytokines in the development of early lesions [34].

Our data suggest that IL-17A blockade could represent a novel therapeutic approach for PGD after transplantation but perhaps also in other detrimental conditions such as pulmonary embolism, ARDS or pulmonary hypertension [35]. Because this work focused on the role of IL-17A, we could not rule out a potential effect for the homologous IL-17F. Indeed, cells producing IL-17A also secrete IL-17F, which both bind the IL-17 receptor as homodimers or heterodimers [36]. Though their structural relationship would suggest similar pro-inflammatory properties, IL‑17F has been shown to rather regulate inflammatory responses [37]. Deciphering the role of IL-17F in solid organ transplantation requires further experiments.

We found no effect of IL-17A deficiency in the development of late lesions of OAD in fully MHC mismatched organs. In contrast, IL-17A blockade could prevent BO in an orthotopic lung transplantation model across minor MHC mismatch [14]. The discrepancies in allogeneic mismatches may explain these results. Indeed, using various skin graft models, we have previously shown that IL17-mediated allograft rejection only occurred in the context of minor antigenic mismatch [38]. Recent data from bleomycin-induced lung injury demonstrated an early IL-17A mediated pulmonary inflammation leading to fibrosis [39,40]. In our post-transplant OAD model, though affording early protection, IL-17A deficiency did not prevent fibrosis, confirming the existence of multiple overlapping fibrotic pathways [41]. In addition, sustained allorecognition of multiple MHC disparities may lead to IFN-γ producing Th1 cells and CTL responses which might hide the early IL-17A-dependent mechanisms [42].

In conclusion, our work demonstrated the direct implication of IL-17A as a pro-inflammatory mediator of early post-transplant airway lesions, which could represent a therapeutic target for prevention of PGD in clinical transplantation.

## Supporting Information

Figure S1 IL-17A, IL-1β, IFN-γ and TNF-α mRNA levels in syngeneic and allogeneic transplants. mRNAs were measured in syngeneic BALB/C or fully allogeneic C57BL/6 tracheas harvested from control or anti-IL17 treated BALB/C recipients after 5 days of transplantation. **A**, IL-17A mRNA. **B**, IL-1β mRNA. **C**, IFN-γ mRNA. **D**, TNF-α mRNA. The bars represent the mean ± SEM of 10 organs in each group. *, p < 0,05.
(TIF)

Figure S2 NK1.1^+^ GILs do not secrete IL-17A. IL-17 producing GILs analysis of fully allogeneic B/C tracheas grafted into B6 recipients and harvested after 8 days. The plots represent the expression of IL‑17A^+^ and H-2K^b+^ GILs. A, the plot is gated on NK1.1^+^ CD3^-^ cells. B, the plot is gated NK1.1 CD3^+^ cells. The plots are representative of 5 organs.
(TIF)
