# Supplementary figures and images for: IL-17A Mediates Early Post-Transplant Lesions after Heterotopic Trachea Allotransplantation in Mice
Source: PLoS One. 2013 Jul 30;8(7):e70236. doi: 10.1371/journal.pone.0070236 (PMC3728020; doi:10.1371/journal.pone.0070236)

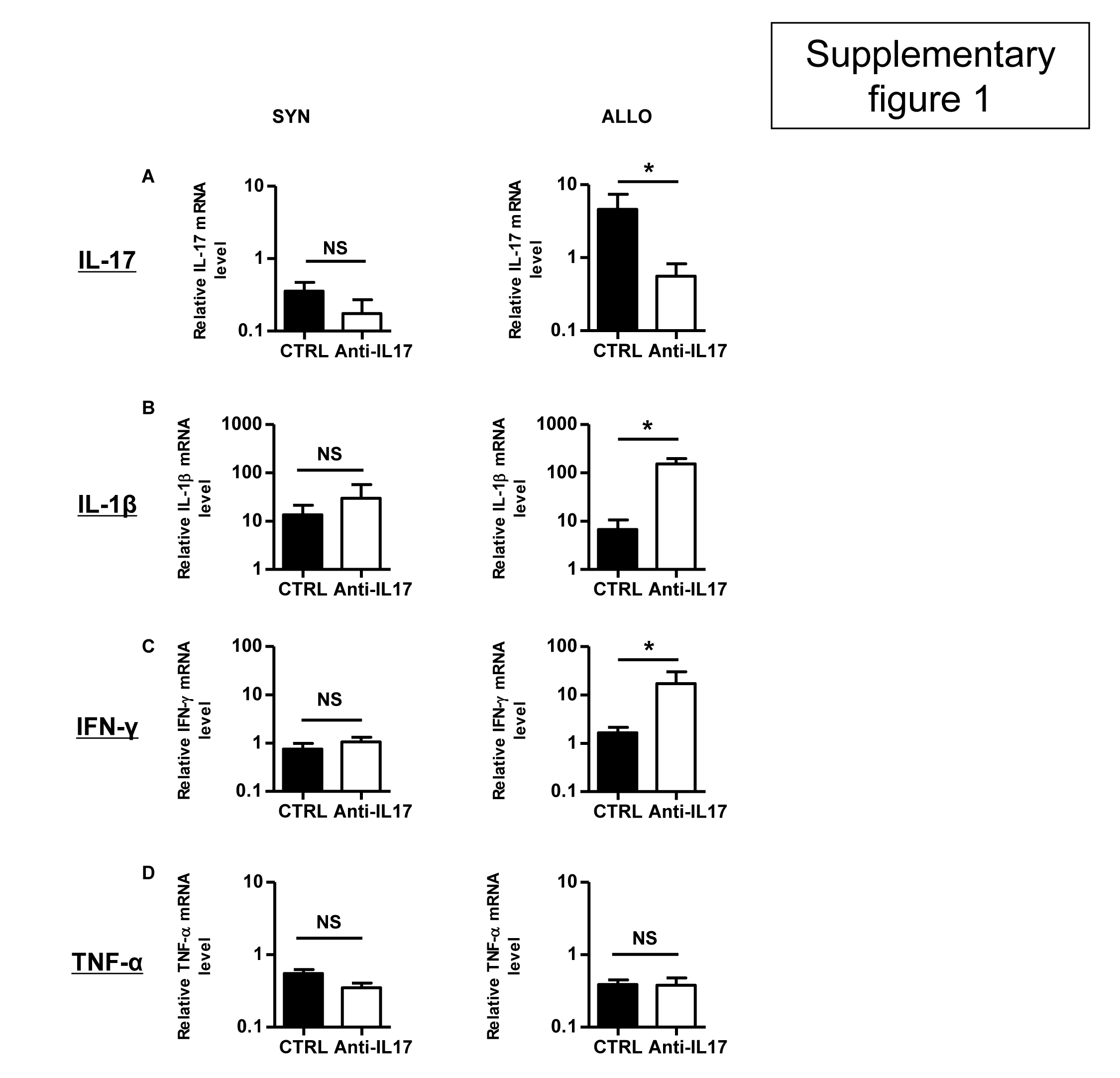

Supplement: Figure S1 — mRNAs were measured in syngeneic BALB/C or fully allogeneic C57BL/6 tracheas harvested from control or anti-IL17 treated BALB/C recipients after 5 days of transplantation. A, IL-17A mRNA. B, IL-1β mRNA. C, IFN-γ mRNA. D, TNF-α mRNA. The bars represent the mean ± SEM of 10 organs in each group. *, p < 0,05. (TIF) [file pone.0070236.s001.tif]

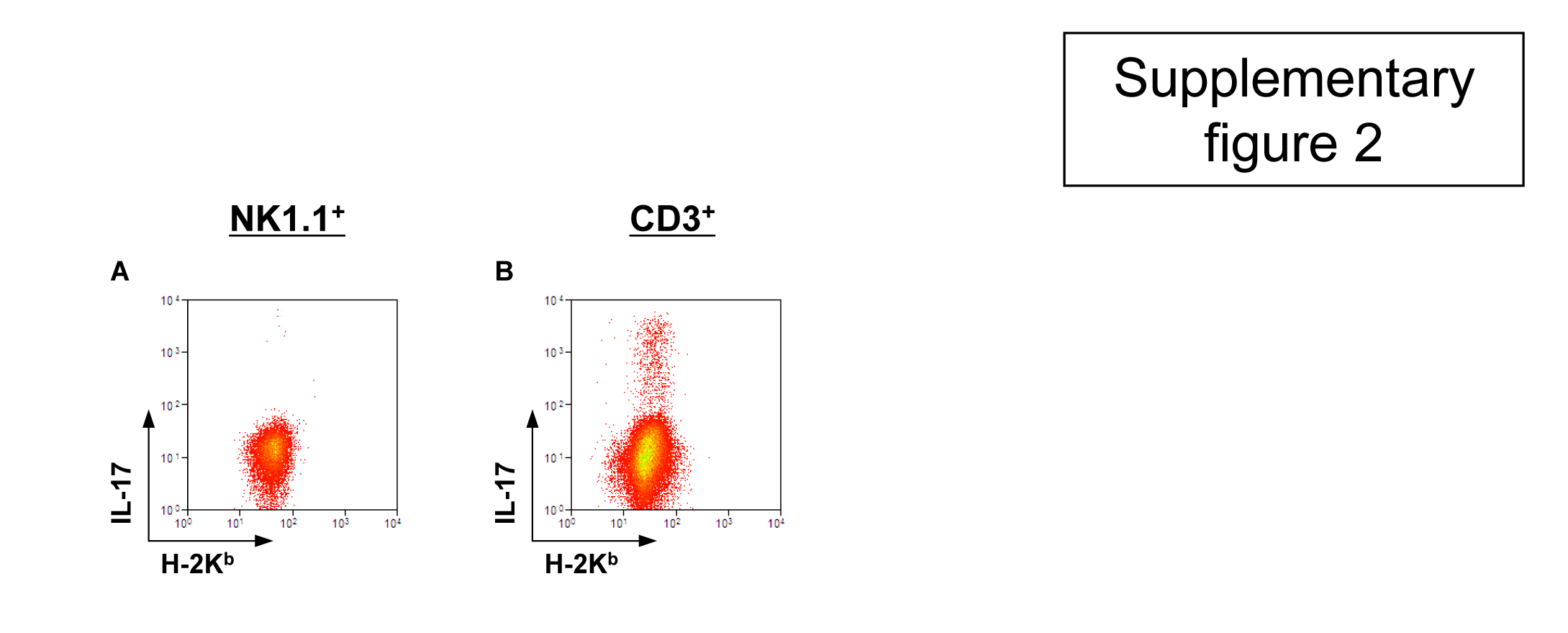

Supplement: Figure S2 — IL-17 producing GILs analysis of fully allogeneic B/C tracheas grafted into B6 recipients and harvested after 8 days. The plots represent the expression of IL‑17A+ and H-2Kb+ GILs. A, the plot is gated on NK1.1+ CD3- cells. B, the plot is gated NK1.1 CD3+ cells. The plots are representative of 5 organs. (TIF) [file pone.0070236.s002.tif]
